# The Dependence of Renal ^68^Ga[Ga]-DOTATOC Uptake on Kidney Function and Its Relevance for Peptide Receptor Radionuclide Therapy with ^177^Lu[Lu]-DOTATOC

**DOI:** 10.3390/diagnostics11071216

**Published:** 2021-07-06

**Authors:** Falk Gühne, Alexander Heinzig, Philipp Seifert, Robert Drescher, Martin Freesmeyer

**Affiliations:** Clinic of Nuclear Medicine, Jena University Hospital, Am Klinikum 1, 07747 Jena, Germany; falk.guehne@med.uni-jena.de (F.G.); alexander.heinzig@uni-jena.de (A.H.); philipp.seifert@med.uni-jena.de (P.S.); robert.drescher@med.uni-jena.de (R.D.)

**Keywords:** DOTATOC, PET/CT, PRRT, kidney function

## Abstract

Background: In addition to its SSTR-specific binding in tumors and healthy tissues, DOTATOC analogues accumulate in kidney parenchyma. Renal tracer uptake might be a surrogate of kidney function or dysfunction. This study aimed to evaluate if kidney function can be estimated from ^68^Ga[Ga]-DOTATOC uptake in PET/CT and its impact on the nephrotoxicity of ^177^Lu[Lu]-DOTATOC PRRT. Methods: Two cohorts of patients (A: 128 diagnostic patients; B: 32 PRRT patients) were evaluated retrospectively. SUV values of the kidneys, physiologically SSTR-expressing organs and in background compartments were assessed. Kidney function was calculated as eGFR by CKD-EPI creatinine equation. Pearson’s correlation coefficients and treatment-induced changes of uptake and kidney function were assessed and compared. Results: Kidney function and renal DOTATOC uptake showed a significant inverse correlation (R^2^ = 0.037; *p* = 0.029). Evaluated models of PET/CT measurements were not able to predict kidney function sufficiently. The uptake of other organs did not depend on eGFR. While the renal uptake increased after PRRT (*p* < 0.001), the kidney function did not change significantly (*p* = 0.382). Neither low pre-therapeutic eGFR nor high pre-therapeutic kidney uptake were risk factors of PRRT-induced deterioration in kidney function. Conclusion: The relevance of kidney function for renal ^68^Ga[Ga]-DOTATOC uptake is limited. The nephrotoxicity of ^177^Lu[Lu]-DOTATOC PRRT might be low and cannot be reliably predicted by pre-therapeutic measurements.

## 1. Introduction

Neuroendocrine tumors (NET), a rare group of tumors originating from the neural and endocrine cells, are mostly located in the gastrointestinal system or the lung [1]. NET typically overexpress somatostatin receptors (SSTR). Radiolabeled somatostatin analogues are established radiopharmaceuticals in diagnostics and the treatment of NET and especially target the SSTR subtype 2 [2]. During the last 20 years, ^68^Ga-DOTA conjugates became widely available and superseded ^111^In-octreotides in tumor detection rate and renal uptake [3]. Nowadays, they are widely used for positron emission tomography/computed tomography (PET/CT) diagnostics in different issues such as tumor staging, or pre- and post-therapeutic evaluation. In addition to classical NET of the pancreas or bowel, PET/CT with ^68^Ga-DOTA conjugates is also able to identify manifestations of rare and extraordinary diseases [4]. In a theranostic approach, beta-emitter-labeled DOTA conjugates can be used for peptide receptor radionuclide therapy (PRRT) for metastasized NET, as recommended by guidelines in different clinical scenarios among other treatment options [5,6]. PRRT aims for a targeted irradiation of SSTR-positive tumor lesions. Nephrotoxicity is a well-known consequence of PRRT and dose-limiting in performing the treatment [7,8,9]. Despite the different affinity with the SSTR subtypes, the DOTA conjugates DOTATOC, DOTATE and DOTANOC show similar results in diagnostic sensitivity and specificity and are equally recommended [10,11].

Beyond their selective binding to NET, ^68^Ga-DOTA conjugates show an accumulation in organs with physiologic SSTR expression, such as the pituitary gland, thyroid, spleen and adrenal glands [12]. Additionally, a more unspecific distribution and excretion leads to a partially or fully SSTR-independent tracer uptake in different tissues, compartments, or organs [3]. The different DOTA conjugates also differ in their biodistribution, which may influence PRRT and its side effects [13]. ^68^Ga-DOTA conjugates are almost exclusively eliminated renally [14]. In addition to the visible urinary excretion in PET/CT diagnostics, the kidney parenchyma shows one of the highest tracer uptakes of all organs. The uptake is mainly located within the inner zone of the renal cortex. Like other small peptides, ^68^Ga-DOTA conjugates undergo glomerular filtration and subsequent reabsorption in the proximal tubules [15]. The reabsorption in tubular cells is primarily mediated by the megalin/cubulin complex, which are endocytic receptors internalizing peptides and thereby modulating the urinary protein excretion [9,16]. Moreover, certain parts of the kidney parenchyma, such as glomeruli, tubule cells and the vasa recta, express SSTR 2 as well [17].

Renal tracer uptake can be a goal or an undesirable side effect of nuclear medicine diagnostics. On the one hand, particular radiopharmaceuticals, e.g., ^99m^Tc[Tc]-DMSA or ^99m^Tc[Tc]-MAG3, enable assessment of the kidney function. On the other hand, renal uptake of radiopharmaceuticals such as ^90^Y- or ^177^Lu-DOTA conjugates may cause irradiation induced renal damage as a potential risk of PRRT [8].

Kaewput and Vinjamuri proposed the hypothesis that rising renal ^68^Ga[Ga]-DOTANOC uptake is a surrogate of kidney dysfunction in patients receiving ^90^Y[Y]-DOTATATE, potentially restricting the application of PRRT [18]. However, the correlation of kidney function and renal ^68^Ga[Ga]-DOTATOC uptake and its relevance for ^177^Lu[Lu]-DOTATOC PRRT is previously undisclosed. Therefore, the aim of this study was to investigate the following questions dedicatedly for DOTATOC radiopharmaceuticals: Does a correlation between renal tracer uptake and kidney function exist? Can the kidney function be determined solely from the PET parameters? Does PRRT influence the kidney function, or solely the renal tracer uptake? Is it possible to predict the potential loss of function from pre-therapeutic tracer uptake?

## 2. Materials and Methods

A retrospective analysis of clinical data collected in our institution from treatment of patients receiving ^68^Ga[Ga]-DOTATOC PET/CT is presented. All diagnostics and therapies were clinically indicated without study-specific requirements. To assess the above-mentioned questions, two different collectives of patients were included in the study.

To investigate the correlation between kidney function and tracer uptake, we assessed all patients receiving a ^68^Ga[Ga]-DOTATOC PET/CT in 2018 (cohort A). A database query resulted in 230 PET/CT examinations. Cases were excluded from the analysis due to insufficient laboratory determination of kidney function (results missing or older than 30 days before PET/CT examination; *n* = 30), PET/CT-protocols only covering parts of the body (*n* = 3), patients undergoing PRRT prior to PET/CT (*n* = 47), and same patients receiving a further PET/CT examination in the period of investigation (*n* = 22).

To examine the influence of PRRT, a longer period of time for data acquisition was assessed (cohort B). A database query of 2013 to 2018 resulted in 60 patients receiving between 1 and 7 cycles of PRRT using ^177^Lu[Lu]-DOTATOC. To ensure the comparability of the results, ^68^Ga[Ga]-DOTATOC PET/CT was assessed before and after 3 cycles of PRRT, which was the standard treatment course within the investigated time period. Patients were excluded if they received less than 3 cycles of PRRT (*n* = 19), did not receive PET/CT after third cycle (*n* = 5), PET/CT only covering parts of the body (*n* = 3), and if PRRT was performed intraarterially (*n* = 1). No time limit was set for the interval between PET/CT and eGFR assessment, due to the low number of patients and the content of the evaluation. PRRT cycles above 3 and respective PET/CT examinations were not included. All included data were acquired before potential further cycles.

The PET/CT scans were performed according to guidelines with an administered activity of approximately 185 MBq ^68^Ga[Ga]-DOTATOC, uptake time was at least 45 min, scanning region from vertex to mid-thigh, iterative reconstruction technique, and scanning time of 2 min per bed position. All examinations were performed with a Biograph mCT40 (Siemens, Germany), a low-dose CT was used for attenuation correction.

The PRRT was performed after interdisciplinary indication, a standard activity of 7.4 GBq ^177^Lu[Lu]-DOTATOC was administered per cycle at our inpatient clinic. Doses were decreased if clinically necessary (e.g., previously known renal impairment). All patients received a standardized protocol of nephroprotective amino acid infusion containing arginine and lysine over 4 h, given parallel to radiotracer application. All patients underwent PET/CT before first cycle for therapy planning and 3 months after the third cycle for response evaluation. PRRT cycles were performed with an interval of 3 months. Interim PET/CT investigations were not taken into account in this study. All patients underwent kidney function determinations by ^99m^Tc[Tc]-MAG3 scintigraphy and ^99m^Tc[Tc]-DTPA scintigraphy prior to therapy.

For estimation of kidney function the glomerular filtration rate (eGFR) was calculated using the CKD-EPI creatinine equation, since this formula outperformed the former equations MDRD and Cockcroft–Gault [19]. For calculating eGFR, serum creatinine level, sex and skin color were considered according to the formula. Additionally, pre-therapeutic tubular excretion rate of ^99m^Tc[Tc]-MAG3 (MAG3-TER) and glomerular filtration rate of ^99m^Tc[Tc]-DTPA (DTPA-GFR) were used for comparison in cohort B.

**Figure 1:**
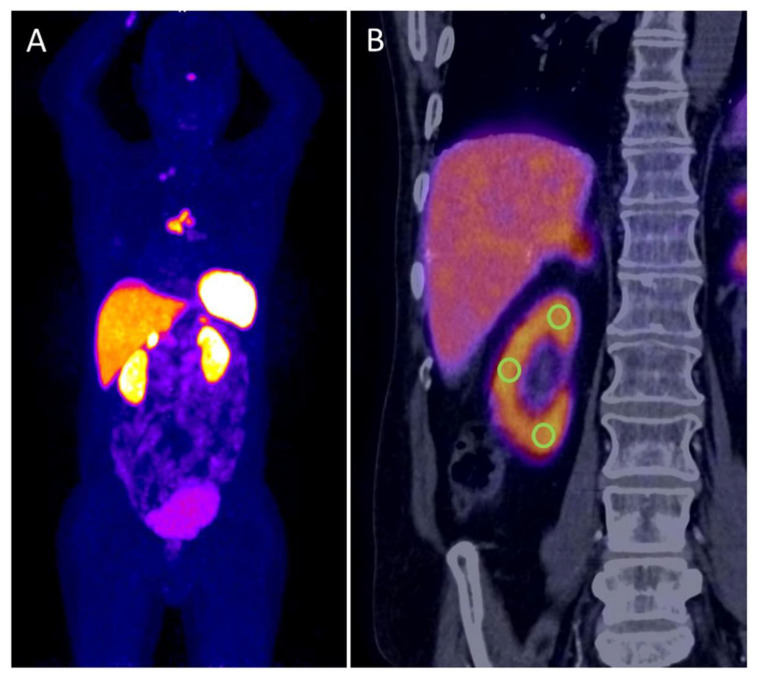
Maximum intensity projection of ^68^Ga[Ga]-DOTATOC PET with high tracer uptake in intrathoracic tumor manifestations, kidneys and typical SSTR-expressing organs (**A**). PET/CT coronal view of right kidney is showing the uptake in renal cortex and the localization of the three defined VOIs for SUV_mean_ measurements, which were averaged with measurements of the contralateral kidney (**B**).

To avoid interobserver variability, only one examiner experienced in PET/CT diagnostics measured renal tracer uptake. Dedicated PET/CT evaluation was conducted without knowledge about kidney function. Standardized uptake values (SUV) were determined at the renal parenchyma, identified with anatomical CT information. Per kidney, the SUV_max_ was assessed, as well as the SUV_mean_ of three defined volumes of interest (VOI) with a diameter of 1 cm at the upper and lower kidney pole, and the lateral aspect of the kidney (Figure 1). For comparison with the global kidney function, the mean value was then calculated from the SUV_max_ and the three SUV_mean_ for both kidneys, respectively. Additionally, tracer concentraction in the urine was measured in both renal pelvis and the urinary bladder (SUV_max_). For comparison, extrarenal organs were quantified. SUV_max_ of typical DOTATOC-positive organs, i.e., pituitary gland, thyroid, spleen and adrenal glands were acquired. Additionally, the SUV_mean_ of surrogates of background compartments were measured in the blood (ascending aorta), muscle (gluteal musculature), liver (parenchyma) and bone (4th lumbar vertebral body). In all measurements, care was taken to ensure that no tumor manifestations were included in the VOI. The individual tumor burden was categorized visually as none, low (less than 5 tumor lesions) or high (5 or more tumor lesions).

Pearson’s correlation coefficient (PCC) was used for statistical analysis of covariance of two variables. The comparison of pre- and post-therapeutic variables was performed by paired *t*-test. The influence of tumor burden was tested by ANOVA with Tukey’s range test. The level of significance was set at 0.05.

## 3. Results

128 patients were included in the evaluation for diagnostic purposes (cohort A). Diagnoses and indications for PET/CT are shown in Table 1. In the evaluation of those patients, we focused on the relationship of renal tracer uptake and kidney function. Mean eGFR was 78.9 ± 23.4 mL/min/1.73 m^2^ (range 9.8–126.2 mL/min/1.73 m^2^), mean SUV_mean_ of kidneys was 11.7 ± 2.9 (range 2.6–21.5) and mean SUV_max_ of kidneys was 18.4 ± 5.0 (range 4.9–31.5). Time interval between laboratory estimation of kidney function and PET/CT was 4.3 ± 6.5 days (range 0–29 days). Mean administered activity was 183.7 ± 6.6 MBq ^68^Ga[Ga]-DOTATOC (range 166–223 MBq), uptake time 61.1 ± 14.7 min (range 42–121 min).

**Figure 2:**
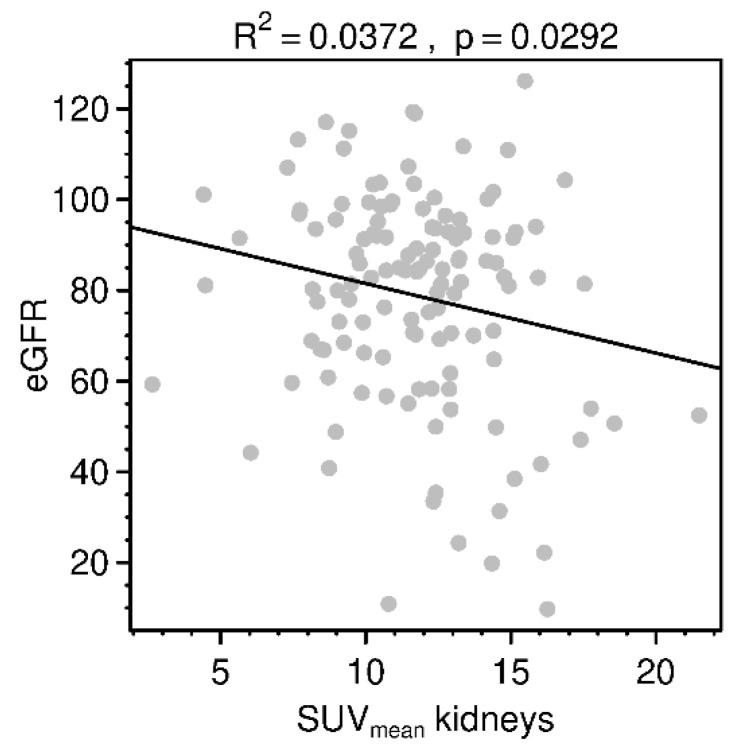
Relationship between eGFR (in mL/min/1.73 m^2^) and SUV_mean_ of the kidneys (both kidneys averaged). Line represents correlation trend. Normal range of eGFR: >90 mL/min/1.73 m^2^.

**Figure 3:**
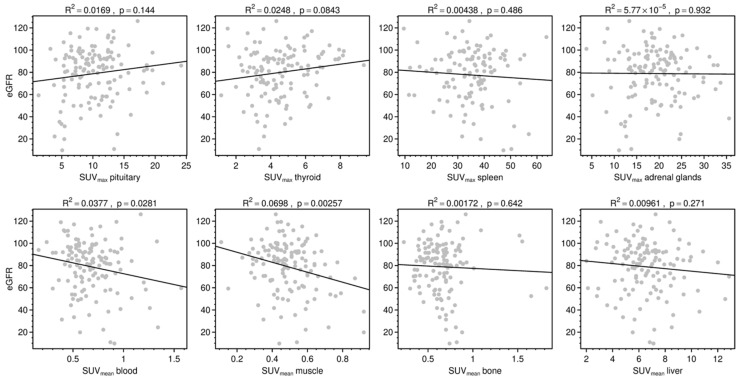
Relationships between eGFR (in mL/min/1.73 m^2^) and SUV_max_ of SSTR-positive organs (upper row) and SUV_mean_ of background compartments (bottom row). Lines represent correlation trends.

No correlation between SUV_max_ of the kidneys and eGFR was found (R^2^ = 0.005; *p* = 0.424). SUV_mean_ showed a significant but weak, negative correlation (R^2^ = 0.037; *p* = 0.029) (Figure 2). By contrast, the uptake of all typically TOC-positive organs showed no correlation to eGFR (Figure 3). Regarding the background tracer distribution, neither SUV_mean_ of the kidney nor eGFR corresponded with bone or liver uptake but showed a significant correlation to blood and muscle uptake (Figure 3). The quotient of uptake in the renal parenchyma and the renal pelvis revealed a moderate correlation with high significance (R^2^ = 0.213; *p* = < 0.001), whereas the quotient of renal parenchyma uptake to urinary uptake in the bladder remained at a very low level of correlation (R^2^ = 0.046; *p* = 0.015). A number of outliers with very high SUV_max_ characterized the uptake measurements of the bladder. Target-background corrections with musculature and with blood (quotient between SUV_mean_ values) had no significant correlations to eGFR (*p* = 0.479; 0.553, respectively). SUV_mean_ of the kidneys was significantly higher within the group with no tumor than in the group with high tumor burden (*p* = 0.029); comparisons of both of those groups with the low tumor burden group were not significant (*p* = 0.945; 0.095, respectively).

Regarding patients who underwent PRRT (cohort B), 32 patients were included (Table 2). This part of the evaluation focused on therapy-dependent changes of renal tracer uptake and eGFR. The mean pre-therapeutic eGFR was 79.8 ± 23.2 mL/min/1.73 m^2^ (range 5.7–111.5 mL/min/1.73 m^2^) and the post-therapeutic eGFR 81.4 ± 22.7 mL/min/1.73 m^2^ (range 7.1–115.8 mL/min/1.73 m^2^). Pre-therapeutic SUV_mean_ of the kidneys was 10.3 ± 2.7 (range 6.1–17.8) and post-therapeutic SUV_mean_ was 12.3 ± 3.9 (range 3.8–23.1), showing an increase of 19%. Mean cumulative administered activity for PRRT was 20.6 ± 3.1 GBq (range 4.1–22.8 GBq). Six patients received less than 20 GBq cumulative dose due to renal impairment, only one of them less than 15 GBq. The mean interval between the third cycle of PRRT and the post-therapeutic eGFR measurement was at 99.1 ± 36.2 days (range 42–204 days), and between the third cycle of PRRT and post-therapeutic PET/CT was 105.6 ± 36.3 days (range 46–211 days).

**Figure 4:**
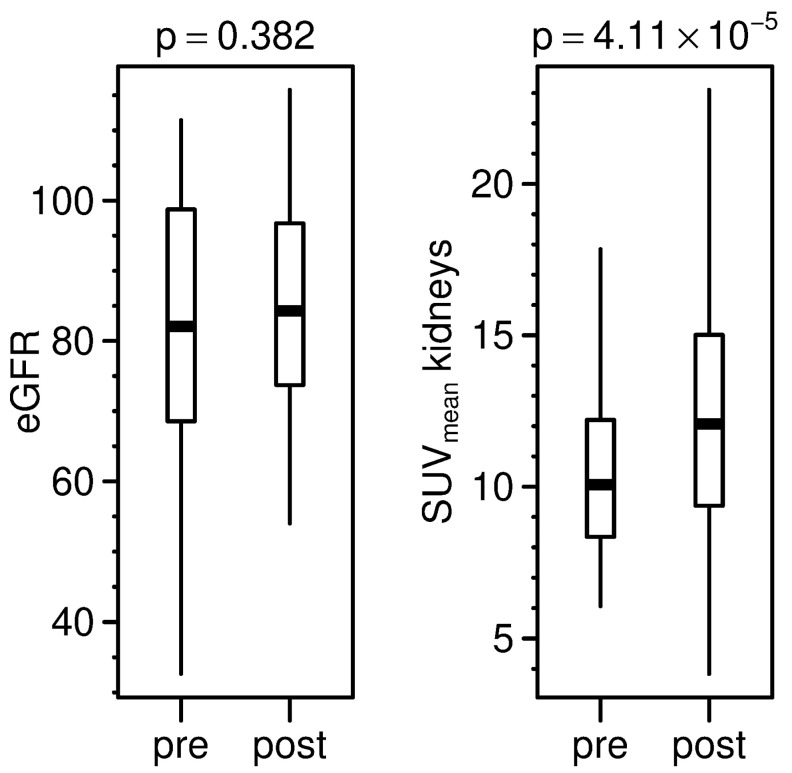
Comparison of pre- and post-therapeutic eGFR (in mL/min/1.73 m^2^) and SUV_mean_ of kidneys.

**Figure 5:**
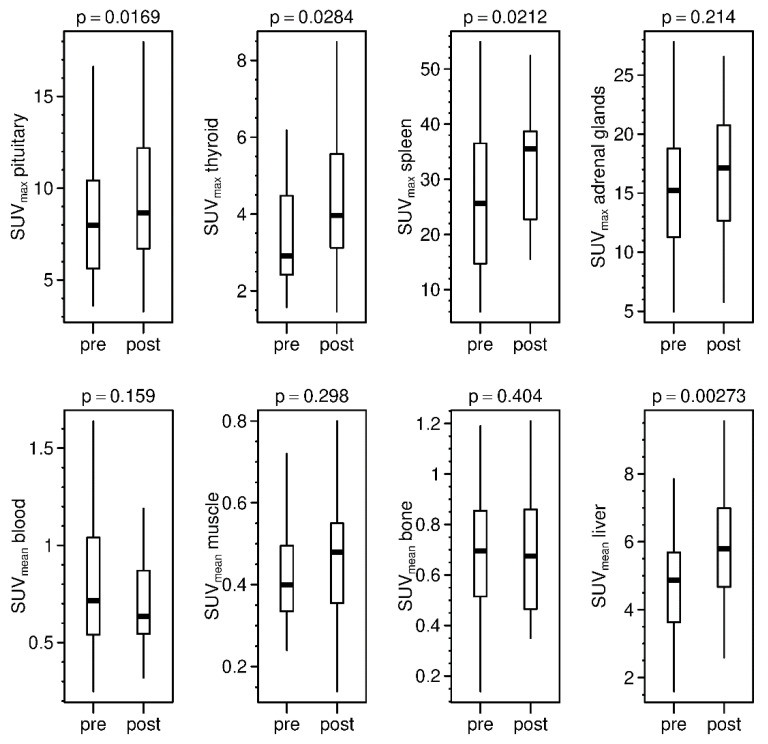
Comparison of pre- and post-therapeutic SUV_max_ of SSTR-positive organs (**upper row**) and SUV_mean_ of background compartments (**bottom row**).

Data revealed a strong intermodal correlation between pre-therapeutic estimations of kidney function, i.e., MAG3-TER compared to eGFR (R^2^ = 0.731; *p* = < 0.001) and DTPA-GFR to eGFR (R^2^ = 0.621; *p* = < 0.001). The SUV_mean_ of kidneys in the pre-therapeutic setting correlated neither with eGFR, nor with MAG3-TER or DTPA-GFR (*p* = 0.356; 0.645; 0.05, respectively). Comparison of the pre- and post-therapeutic SUV_mean_ of the kidneys showed a significant increase in uptake (*p* ≤ 0.001) (Figure 4). However, there was no significant change in eGFR (*p* = 0.382) and no significant correlation between change in SUV_mean_ of the kidneys and change in the eGFR (R^2^ ≤ 0.001; *p* = 0.915). DOTATOC-positive organs showed a significant increase in SUV_max_, except for adrenal glands (*p* = 0.214, tending to increase as well) (Figure 5). Background compartments showed, in contrast, no significant change due to PRRT, except for liver (*p* = 0.002), which increased in post-therapeutic PET/CT (Figure 5). Overall, there were inconsistent dynamics of SUVs and eGFR with individually increasing and decreasing values.

**Figure 6:**
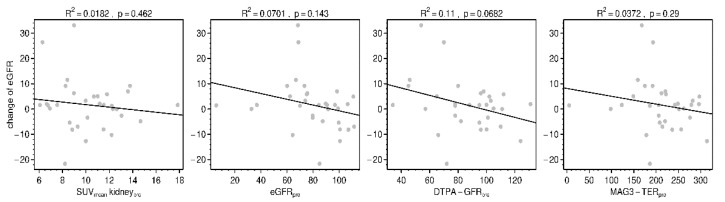
Relationships between PRRT-associated change of eGFR (in mL/min/1.73 m^2^) and pre-therapeutic measurements: SUV_mean_ of kidneys, eGFR (in mL/min/1.73 m^2^), DTPA-GFR and MAG3-TER (both in mL/min). Lines represent correlation trends.

The PRRT-associated change of eGFR did not correlate significantly with pre-therapeutic kidney uptake, with pre-therapeutic renal function (eGFR, DTPA-GFR, MAG3-TER) or with the cumulated administered dose over all three cycles of PRRT (Figure 6).

Even highly deficient kidneys, such as in one patient with high-grade renal insufficiency requiring dialysis and a second patient with one non-functioning kidney, showed a ^68^Ga[Ga]-DOTATOC uptake only slightly lower than in normal functioning kidneys, with average pre-therapeutic SUV_mean_ of 7.2 and post-therapeutic SUV_mean_ of 7.8, indicating a treatment-dependent increase of 8%. There was no correlation between the partial function of one kidney in MAG3 scintigraphy and partial SUV_mean_ of the same kidney in the pre-therapeutic setting (*p* = 0.605).

## 4. Discussion

A significant negative, albeit weak correlation between kidney function and renal tracer uptake exists: the lower the eGFR, the higher the renal uptake of ^68^Ga[Ga]-DOTATOC. Therefore, kidney function appears not to be a major influencing factor in DOTATOC uptake in kidney parenchyma and other parameters have to be co-factors. Correlation would have been slightly stronger if only laboratory eGFR assays within 3 days prior to PET/CT had been considered. Even within a time interval of 30 days kidney function could vary significantly. In addition, a difference in hydrogenation status can have a relevant and even more rapid effect. The methodical inaccuracy of creatinine-based eGFR may reduce the significance of correlation in general. The uptake time between the administration of ^68^Ga[Ga]-DOTATOC and the scan is a significant confounder to the correlation in our study. Nevertheless, the correlation coefficient would not substantially increase by this secondary exclusion or correction (R^2^ = 0.067; 0.043, respectively). Additionally, the tumor burden had a weak but significant influence on renal uptake in our patients, possibly indicating a steal effect by the high tracer avidity of the NET tissue. The existence of a so-called tumor sink effect has been discussed, controversially, in this setting [20,21]. A former splenectomy, also found in a few patients in our cohort, has a previously reported effect on renal uptake [22]. Further factors such as age and gender, as well as secondary diseases such as diabetes or former nephrotoxic treatments, which were not ascertained here, may be of influence [23]. Despite glomerular filtration, ^68^Ga[Ga]-DOTATOC is not suitable for determining kidney function due to the tubular absorption and, above all, the specific extrarenal binding in tumor tissue and organs.

Since the uptake of other physiologically DOTATOC-positive organs, like the pituitary gland, thyroid, spleen and adrenal glands, does not correlate with kidney function, a general dependency on SSTR-specific binding can be excluded. The effect of the kidney function on the tracer allocation to the blood and muscle compartments might be caused by decreased renal tracer excretion due to loss of eGFR. The effect of kidney function seems to be similar for renal, blood and muscle uptake. Therefore, correction of renal uptake with blood or muscle uptake had a negative effect on correlation to kidney function. Dedicated models were evaluated, aiming to achieve the highest possible correlation between renal uptake and kidney function, whereby the quotient from parenchymal uptake to urinary uptake within the renal pelvis had a superior performance, potentially representing a dynamic function of excretion. The urinary SUV measurement within the renal pelvis is more feasible than in the bladder, in which a large amount of outlier values influences the results.

Within the population of patients undergoing therapy, the weak correlation between kidney function and renal uptake could not be verified, due to the small size of the cohort. This applies to the eGFR by CKD-EPI as well as the MAG3-TER and the DTPA-GFR. A high agreement between pre-therapeutic eGFR and MAG3-TER or DTPA-GFR confirmed the validity of the kidney function determinations and is in accordance with previous study results [24,25].

The comparison between pre- and post-therapeutic eGFR showed no significant reduction. PRRT, especially if performed with ^177^Lu[Lu]-DOTATOC, might not be as nephrotoxic as assumed, which was suggested by other authors as well [26]. Possibly, the previously reported decrease in eGFR is caused by ^90^Y-labeled SSTR-analogues only [18], which were shown to be more nephrotoxic than ^177^Lu-radiopharmaceuticals [13,27]. The prophylactic application of amino acid infusions is effective in protection of the kidneys [5,28] and may prevent nephrotoxicity mostly at administered single doses of up to 7.4 GBq. On the other hand, it cannot be ruled out that renal insufficiency may appear later, after a progressive deterioration in kidney function has been demonstrated for months and years after the PRRT [28]. The post-therapeutic eGFR of our study was assessed, approximately, only 3 months after the third cycle, but 9 months after the first cycle of the PRRT; therefore, a considerable interval had passed. eGFR might be the wrong parameter to reveal kidney dysfunction, as mainly the proximal tubules were irradiated and thus nephrotoxicity could be underestimated [27,28], but, also, post-therapeutic MAG3-TER and DTPA-GFR, which were only available in some of our patients, failed to verify renal dysfunction. The differences between our results and the results of Kaewput and Vinjamuri could be caused by different SSTR-analogues being used for PET/CT. Nevertheless, renal uptake of DOTATOC and DOTATE is proposed to be similar [29,30].

Dosimetry of the kidneys is possible but challenging [31,32]. In particular, the PET/CT-based pre-therapeutic estimation of kidney dose in PRRT is limited due to the short physical half-life of ^68^Ga, which cannot depict the biokinetics of ^177^Lu during treatment [21]. Consequently, the pre-therapeutic PET and laboratory data in this study were not able to predict a change of kidney function [26]. Although insignificant, the patients with high pre-therapeutic kidney function (in all three assessments) tended towards a minor worsening of eGFR by PRRT, which lowers concerns about a further deterioration in the case of initial kidney insufficiency. Even though the increase in kidney uptake as a result of PRRT was significant, no correlation between change in uptake and change in kidney function could be verified. However, it is possible that the increased renal uptake could be a predictor or early sign of a later deterioration in kidney function. A compensatory increase in megalin, caused by defects of tubular cells as described in elderly humans [33], might be the reason for increased uptake. Although a high renal uptake correlates overall with poorer kidney function, functionless kidneys in particular showed a relatively low uptake and a lower therapy-associated increase in uptake. A purely mathematical relationship between deterioration in kidney function and increasing renal DOTATOC uptake thus appears unlikely. In contrast to previous studies, a few other organs also showed an increased uptake [34]. Hence, it cannot be ruled out that an elevated tracer availability in general, caused by tumor regression in consequence of PRRT, is the decisive factor.

Method discussion and limitations: To validate a mathematic correlation between kidney function and renal tracer uptake a bigger sample with variability in kidney function was required, since the population of patients receiving PRRT was relatively small in our study, as in other studies before [18,20,26]. Therefore, a second collective of patients, independent of PPRT had to be assessed. We assume that their results can, in principle, be transferred to PRRT patients. Due to retrospective evaluation, missing or time-variable parameters could have influenced the validity of our study. Nevertheless, by performing PET/CT and PRRT consequently complying with institutional standard operating procedures, as well as the dedicated review of all 192 PET/CT by one examiner, the variability could be kept low. Individually drawn VOIs to assess the SUVmean of entire kidney parenchyma, used in other studies [18], could have increased the reliability of measurements, but the concordance of the three exemplary VOIs was high, which were therefore deemed to be representative. Computer-assisted rendering of voxel-based isocontours or isosurfaces is ineligible due to high SUV in the renal pelvis or adjacent organs such as the spleen.

## 5. Conclusions

The relationship between kidney function and renal ^68^Ga[Ga]-DOTATOC uptake is inversely proportional but of negligible importance. Kidney function has a significant impact on biodistribution, but it is not the only variable. It is therefore not possible to reliably estimate kidney function based on PET measurements alone. Nephrotoxicity of ^177^Lu[Lu]-DOTATOC PRRT is limited, an early deterioration in kidney function was not seen, but renal tracer uptake increased significantly. Therefore, rising renal uptake cannot be ruled out to be a pre-sign of renal malfunction. Estimation of nephrotoxicity remains limited since pre-therapeutic renal uptake or function could not predict a therapy-induced change of kidney function.

## Figures and Tables

**Table 1:** Patient characteristics of cohort A.

***n*, Sex**	128 (57 female/71 male)	
**Age**	61.8 ± 13.1 years	
**Diagnosis**	foregut NET:	35
	midgut NET:	22
	hindgut NET:	6
	NET of unknown primary:	5
	lung carcinoid:	10
	unconfirmed suspicion:	28
	others:	22
**Tumor Load**	none:	66
	low:	37
	high:	25
**eGFR**	>90 mL/min/1.73 m^2^:	48
	60–90 mL/min/1.73 m^2^:	52
	<60 mL/min/1.73 m^2^:	28

**Table 2:** Patient characteristics of cohort B.

***n*, Sex**	32 (16 female/16 male)	
**Age**	64.2 ± 11.1 years	
**Diagnosis**	foregut NET: midgut NET: NET of unknown primary: others:	16 6 4 6
**Cumulative Dose**	20.7 ± 3.7 GBq	
**Pre-Therapeutic eGFR**	>90 mL/min/1.73 m^2^: 60–90 mL/min/1.73 m^2^: <60 mL/min/1.73 m^2^:	12 17 3
